# Unusual Electrical Transport Driven by the Competition between Antiferromagnetism and Ferromagnetism in Antiperovskite Mn_3_Zn_1−x_Co_x_N

**DOI:** 10.3390/ma11020286

**Published:** 2018-02-12

**Authors:** Lihua Chu, Lei Ding, Cong Wang, Meicheng Li, Yanjiao Guo, Zhuohai Liu

**Affiliations:** 1 State Key Laboratory of Alternate Electrical Power System with Renewable Energy Sources, School of Renewable Energy, North China Electric Power University, Beijing 102206, China; mcli@ncepu.edu.cn (M.L.); guoxiaojiaooo@163.com (Y.G.); zhuohaiLiu666@163.com (Z.L.); 2 ISIS Neutron and Muon Source, Rutherford Appleton Laboratory, Harwell Oxford, Didcot OX11 0QX, UK; 3 Center for Condensed Matter and Materials, Department of Physics, Beihang University, Beijing 100191, China; congwang@buaa.edu.cn

**Keywords:** magnetic properties, electrical properties, negative thermal expansion, antiperovskite

## Abstract

The magnetic, electrical transport and thermal expansion properties of Mn_3_Zn_1−x_Co_x_N (x = 0.2, 0.4, 0.5, 0.7, 0.9) have been systematically investigated. Co-doping in Mn_3_ZnN complicates the magnetic interactions, leading to a competition between antiferromagnetism and ferromagnetism. Abrupt resistivity jump phenomenon and negative thermal expansion behavior, both associated with the complex magnetic transition, are revealed in all studied cases. Furthermore, semiconductor-like transport behavior is found in sample x = 0.7, distinct from the metallic behavior in other samples. Below 50 K, resistivity minimum is observed in samples x = 0.4, 0.7, and 0.9, mainly caused by e-e scattering mechanism. We finally discussed the strong correlation among unusual electrical transport, negative thermal expansion and magnetic transition in Mn_3_Zn_1−x_Co_x_N, which allows us to conclude that the observed unusual electrical transport properties are attributed to the shift of the Fermi energy surface entailed by the abrupt lattice contraction.

## 1. Introduction

As a strongly correlated electron system, antiperovskite compounds with a chemical formula Mn_3_XN (X: transition metals or semiconducting elements) and with a noncollinear magnetic ground state induced by the geometric frustration in the Mn_6_N octahedron have been shown to exhibit fascinating physical properties, such as abnormal thermal expansion including negative thermal expansion (NTE) and zero thermal expansion etc. [1,2,3], near-zero temperature coefficient of resistivity (TCR) [4,5,6], magnetostriction [7], spin-glass (SG) behavior [8,9,10] and magnetocaloric effect [11,12]. It has been found that these interesting physical properties are sensitive to the number of the valence electrons of metal X located at the corners of antiperovskite unit cell, which contributes itinerant electrons at the Fermi level [13]. Hence, any change in carrier concentration of Mn_3_XN has a significant impact on its electronic structure, and may produce great diversity of its magnetic structures and related novel physical phenomena [14]. 

Among these antiperovskite compounds, Mn_3_ZnN with the so-called noncollinear Γ^5g^ antiferromagnetic (AFM) structure, has attracted considerable attention [15,16]. Previous reports have demonstrated that Mn_3_ZnN undergoes an AFM transition at around 180 K, responsible for a resistive-switching phenomenon [17], and two cubic phases appear in a given temperature range [18]. Chemical doping and defect based on Mn_3_ZnN could lead to additional interesting magnetic and electronic properties [1,19,20,21,22]. For example, the zero thermal expansion (ZTE) of Mn_3_[Zn-(Ag,Ge)]_x_N compounds has been investigated, and is closely correlated with the magnetic structure and can be tuned by adjusting the chemical and vacancy concentrations [1] and the crystallite size [23]. Magnetoresistance reversal has been reported in Mn_3_Cu_0.5_Zn_0.5_N, which is thought of as the reconstruction of the Fermi surface accompanied by an AFM- ferromagnetic (FM) transition [24]. Moreover, recent reports have shown that the introduction of Co could effectively tune the physical properties in antiperovskites, such as the near zero TCR in Mn_3−x_Co_x_CuN [25] and the AFM-FM transition in Mn_3_Ag_1−x_Co_x_N [26]. Since Co bears a similar electronic structure to that of Zn, introducing magnetic Co in Mn_3_ZnN may provide new insight into the understanding of the origin of these novel physical properties.

In this study, we present the magnetic, electrical transport and thermal expansion properties of Mn_3_Zn_1−x_Co_x_N compounds. The doping of magnetic Co at the Zn site in Mn_3_ZnN can effectively modify the magnetic interactions and trigger strong AFM and FM competition. The competing interaction may prompt the unusual transport properties in Mn_3_Zn_1−x_Co_x_N.

## 2. Materials and Methods 

### 2.1. Sample Preparation

Polycrystalline samples Mn_3_Zn_1−x_Co_x_N (x *=* 0.2, 0.4, 0.5, 0.7, 0.9) were synthesized by solid-state reaction from stoichiometric mixtures of Mn_2_N, Zn, and Co powders. These powders were carefully mixed and ground in a mortar, and then pressed into pellets. The pellets were wrapped in Ta foils and sealed in vacuum (*p* < 10^−5^ Pa) into quartz tubes. The tubes were then sintered at 1073.15 K for 80 h, and cooled down to room temperature. 

### 2.2. Characterization

Variable-temperature XRD experiments in the temperature range 10–300 K were carried out on a Bruker D8 ADVANCE diffractometer (Bruker Corporation, Billerica, MA, USA ) with *Kα1* radiation selected by a Ge (111) primary beam monochromator. The measurements of the temperature-dependent magnetization from 10 K to 350 K were performed on Magnetic Property Measurement System (MPMS) (Quantum Design, San Diego, CA, USA). Both zero-field-cooled (ZFC) and field-cooled (FC) magnetization curves were measured from 5 K to 350 K under external magnetic field of 600 Oe. Magnetic hysteresis loops between 0 and 5 T were recorded at 50, 100, 150, 200, and 300 K. The electrical resistivity was measured using the standard four-probe method with a commercial (Quantum Design, Inc.) physical property measurement system (PPMS). Differential Scanning Calorimeter (DSC200F3, NETZSCH, Gebrüder-Netzsch-Straße, Selb, Germany) was used to measure the specific heat of the samples in the temperature range 110–300 K. 

## 3. Results and Discussion

### 3.1. Crystal Structure

**Figure 1:**
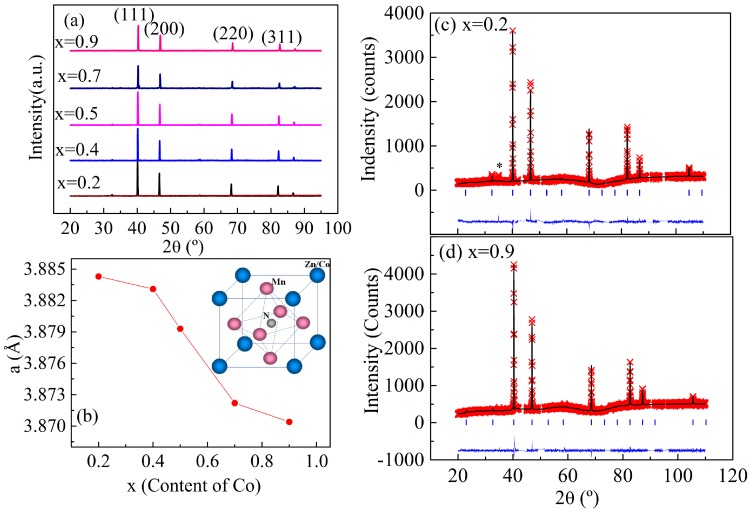
(**a**) Powder XRD patterns of the polycrystalline Mn_3_Zn_1−x_Co_x_N (0 ≤ x ≤ 1) at room temperature; (**b**) Lattice parameter as a function of the Co content. Inset shows the schematic crystal structure of Mn_3_Zn_1−x_Co_x_N; (**c**,**d**) Rietveld analysis of the XRD patterns for Mn_3_Zn_1−x_Co_x_N (x = 0.2 and 0.9) observed at room temperature. The cross marks and solid lines show the observed and calculated patterns, respectively. The difference between them is shown at the bottom of each panel. The positions of the Bragg reflections are marked by ticks. The symbol of asterisk indicates the impurity phase MnO. Excluded regions are the diffraction reflections from sample holder (Cu).

The crystal structures of all samples were investigated using powder XRD at room temperature, as shown in Figure 1a. All samples crystallize in the cubic antiperovskite structure with the space group *Pm*-3*m*. The XRD pattern of all the Mn_3_Zn_1−x_Co_x_N (0 < x ≤ 1) samples were analyzed using the Fullprof software [27]. An initial analysis of all patterns was carried out by assuming a space group *Pm*-3*m* with N, Zn/Co, and Mn atoms at the 1a site (0, 0, 0), 1b site (1/2, 1/2, 1/2), and 3d site (1/2, 0, 0), respectively. The good agreement between the observed and calculated patterns from the Rietveld refinement indicates that the *Pm*-3*m* model is suitable for Mn_3_Zn_1−x_Co_x_N compounds. Figure 1c,d shows the refined results for samples x = 0.2 and 0.9, respectively. A small amount of impurity phase MnO was marked in Figure 1 and was not considered in the refinement. The lattice parameter as a function of the Co concentration is shown in Figure 1b. The lattice constant decreases monotonically with increasing Co content, as the atomic radius of Co is smaller than that of Zn. These results indicate that Co replaces Zn in Mn_3_ZnN as designed.

### 3.2. Magnetic Properties

**Figure 2:**
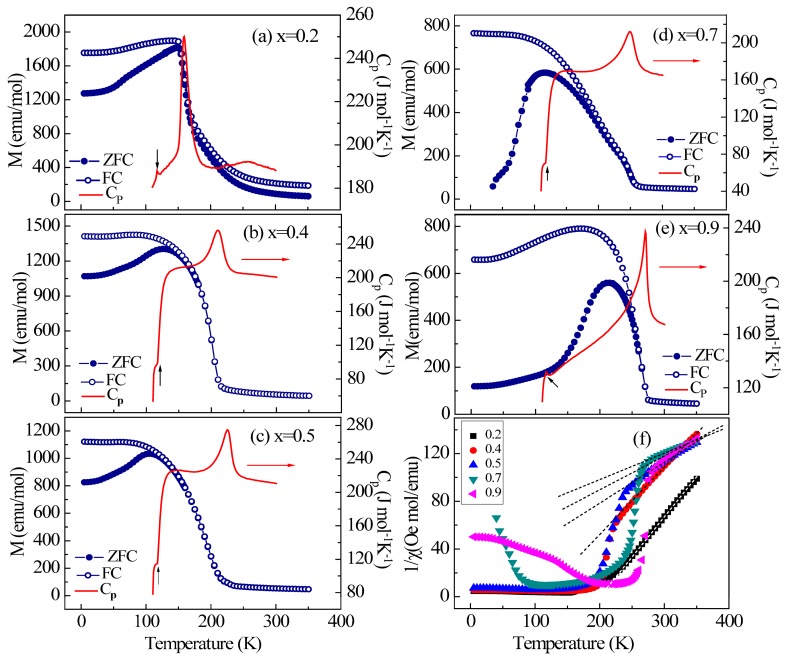
Temperature dependence of the magnetization and specific heat C_P_ for Mn_3_Zn_1−x_Co_x_N compounds: (**a**) x = 0.2; (**b**) x = 0.4; (**c**) x = 0.5; (**d**) x = 0.7; (**e**) x = 0.9. The small cusp in specific heat curves at 115 K represents the contribution of MnO; (**f**) Temperature dependence of the inverse magnetic susceptibility for Mn_3_Zn_1−x_Co_x_N. The fitting lines represent the Curie-Weiss curve.

The temperature dependence of the magnetization curves M (T) of the polycrystalline Mn_3_Zn_1−x_Co_x_N under ZFC and FC processes is shown in Figure 2. In sharp contrast to the host material Mn_3_ZnN where an AFM transition occurs at ~183 K [15], the samples x = 0.2, 0.4, 0.5, 0.7 and 0.9 show a magnetic state featured by a canted AFM magnetic ground state, resulting from the competition between FM and AFM interactions. The irreversibility between ZFC and FC curves probably implies the presence of FM components related to canting phenomenon. It is worth noting that the magnetization decreases as the Co-doping proceeds, indicating the suppression of FM interactions and the enhancement of AFM interactions. In order to further study the magnetic properties, we performed specific heat measurement. All temperature-dependent specific heat C_P_ curves (Figure 2) exhibit an obvious peak around the magnetic transition T_N_, in good accordance with the magnetization results. The small cusp in specific heat curves at ~115 K is a contribution of impurity phase MnO which undergoes a magnetic phase transition at 115 K.

**Figure 3:**
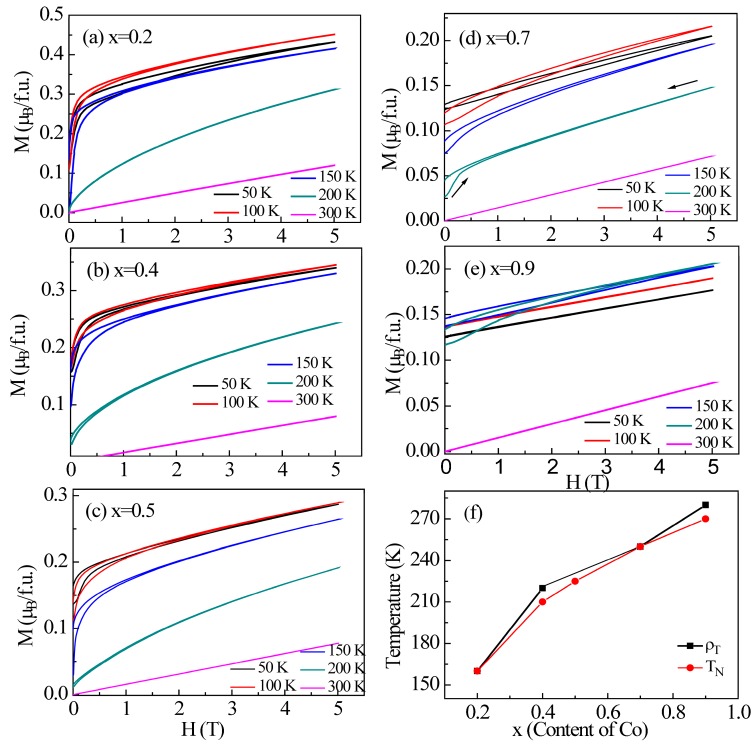
Isothermal magnetization curves M (H) measured from 0 T to 5 T and from 5 T to 0 T at several selected temperatures from 50 to 300 K for Mn_3_Zn_1−x_Co_x_N (**a**) x = 0.2; (**b**) x = 0.4; (**c**) x = 0.5; (**d**) x = 0.7; (**e**) x = 0.9. The measurement process of the M (H) curves are shown in (**d**) by arrows. These curves are not typical magnetic hysteresis loops; therefore, the remnant magnetization should be positive. The idea of this characterization is to evidence the presence of ferromagnetic components in the as-prepared samples. Some initial magnetization curves do not develop from zero, which is caused by the history of the magnetization of the samples; (**f**) Variations of the transition temperature T_N_ and resistivity ρ_T_ as a function of Co content.

To obtain information on the nature of the magnetic order, the spin-only expression: χ(T) = C/(T − *Θ*_W_), where C is the Curie constant and *Θ*_W_ is the Weiss temperature, was applied to fit the paramagnetic linear region of the magnetic susceptibility curves, as shown in Figure 2f. The fitting results for all samples are presented in Table 1. For samples x = 0.2 and 0.4, positive *Θ*_W_ of 200 and 115 K were obtained, respectively, indicating the dominant FM interactions. However, negative *Θ*_W_ were obtained for x ≥ 0.5, suggesting the governing role of the AFM interactions. Such a variation of the sign of *Θ*_W_ implies the enhancement of the AFM interactions with increasing Co concentration. Moreover, the effective magnetic moment μ_eff_ of all the compounds is much lower than the theoretical value of 4 μ_B_/Mn [28], which is consistent with the magnetism from itinerant electrons, as expected for these compounds. As shown in Figure 3a–e, the presence of a small fraction of ferromagnetic components can be confirmed in the isothermal M-H curves. For x = 0.2, the magnetization does not reach saturation, but has a remnant magnetization value of 0.32 µ_B_/f.u. as the magnetic field increases below T_N_. The remnant magnetization decreases with increasing Co content. These features imply that the Mn_3_Zn_1−x_Co_x_N compounds possess a canted AFM magnetic ground state with x > 0. Regarding the magnetic interaction pathways, previous studies have shown consistently that the triangular lattice composed of Mn atoms is uniquely responsible for the magnetic properties. It is hence reasonable to believe that the significant spin interactions arise from Mn-Mn atoms. However, based on our current experimental results, we cannot rule out the possibility of the existence of exchange interactions between Co-Co atoms, which may affect the remnant magnetization. Further verification about this point requires advanced experimental techniques such as neutron diffraction.

### 3.3. Electrical Transport Behavior and Negative Thermal Expansion 

**Figure 4:**
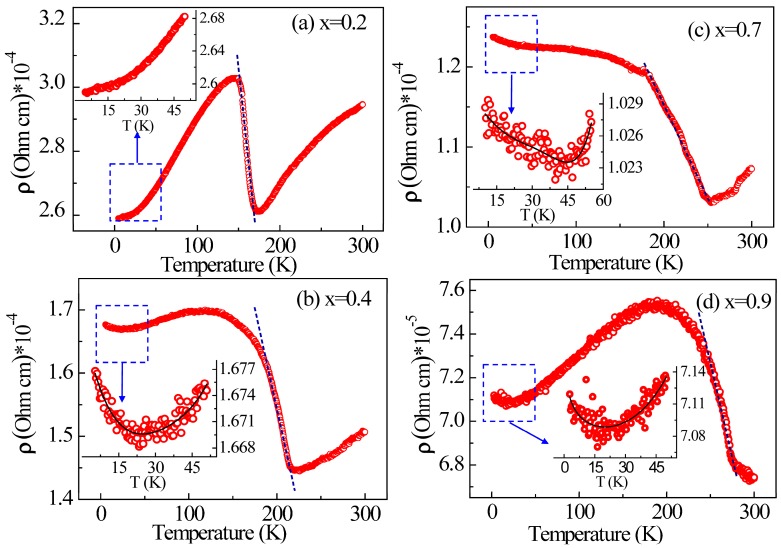
Temperature dependence of ρ for Mn_3_Zn_1−x_Co_x_N on warming for (**a**) x = 0.2; (**b**) x = 0.4; (**c**) x = 0.7; (**d**) x = 0.9. Insets show low-temperature resistivity data plotted and the fitting (solid line) using function (1) at low temperature. The dashed line represents the jumping zone.

Figure 4 shows the temperature-dependent resistivity ρ (T) of Mn_3_Zn_1−x_Co_x_N (x = 0.2, 0.4, 0.7, 0.9) in the temperature range of 5–350 K. No large magnetoresistance behavior was observed in any of the studied samples (see Figure S1 in Supplemental Materials). For x = 0.2, the resistivity first decreases upon cooling from 300 K, then increases abruptly at T_N_ = 175 K, reaching a maximum, and finally decreases with a further decrease in temperature. The resistivity exhibits a temperature-dependent fluctuation up to 20% (evaluated by the function (ρ_max_ − ρ_mim_)/ρ_mim_), which is much higher than that in Mn_3_ZnN [17]. Sample with x = 0.4 also shows abrupt resistivity change at T_N_, but with a very slight decrease after a resistivity maximum. Namely, the transport behavior at low temperature is metallic for Mn_3_Zn_1−x_Co_x_N (x = 0.2 and 0.4) compounds. For x = 0.7, there is an obvious increase in resistivity around T_N_ = 260 K with decreasing temperature down to 175 K, as shown in Figure 4c. Then the resistivity increases slowly but monotonically to the lowest measured temperature. In contrast to x = 0.2 and 0.4, the temperature dependence of the resistivity of Mn_3_Zn_0.3_Co_0.7_N shows a semiconductor-like transport behavior which is likely a result of the change of its energy band structure [29]. This gives rise to a metal-to-semiconductor-like change from sample x = 0.2 to x = 0.7. 

It has been previously shown that Mn_3_ZnN undergoes a resistive switching phenomenon driven by AFM phase separation below 190 K [17]. In our work, the behavior of electrical transport in all investigated samples (from x = 0.2 to 0.9) shows an abrupt resistivity jump around T_N_. Among them, the sample with x = 0.9 exhibits an abrupt resistivity change at the much higher temperature T_N_ = 276 K. In all cases, the abrupt resistivity jump phenomenon is accompanied by magnetic ordering, indicating a strong correlation between the magnetism and the electrical transport. 

**Figure 5:**
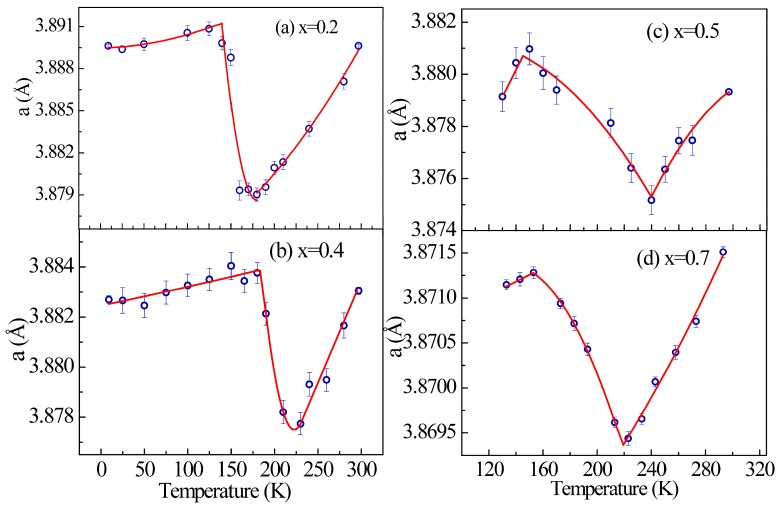
Temperature dependence of the cubic lattice parameter for Mn_3_Zn_1−x_Co_x_N (**a**) x = 0.2; (**b**) x = 0.4; (**c**) x = 0.5; (**d**) x = 0.7. Negative thermal expansion behavior around the magnetic phase transition was observed.

To explain the unusual electrical transport behavior, we investigated the thermal expansion behavior of Mn_3_Zn_1__−__x_Co_x_N series using variable temperature XRD. XRD data collected at different temperatures reveal that all samples crystallize into cubic cells with space group *Pm*-3*m*, and no structural transition can be observed over the whole measured temperature range. The refinement of the XRD data yields the temperature dependence of the lattice constant, as shown in Figure 5. It can be seen that all samples display a negative thermal expansion behavior in their specific temperature range. For samples x = 0.2, 0.4, 0.5, and 0.7, the temperature range could be estimated at about 125–180 K, 180–230 K and 150–240 K, 153–223 K, respectively, with respective linear coefficients of thermal expansion −5.53 × 10^−5^ K^−1^, −3.1 × 10^−5^ K^−1^, −1.67 × 10^−5^ K^−1^ and −0.68 × 10^−5^ K^−1^. Obviously, the introduction of Co effectively broadens the temperature range of NTE. This is because of the negative thermal expansion associated with the magnetic phase transition through the magneto-volume effect, as suggested in several antiperovskites [1,2,3]. On the basis of this lattice change, we may discuss slightly the underlying cause for unusual electrical transport properties in Mn_3_Zn_1−x_Co_x_N. The abrupt change in the lattice parameter, caused by the magnetic transition, may lead to the shift of Fermi level. Therefore, an abrupt decrease in the DOS near the Fermi level could be generated through the shift of the Fermi energy surface, leading to a pronounced decrease of the effective number of conduction electrons, as explained in Ref. [29]. Therefore, the resistivity can be enhanced significantly at the magnetic transition temperature. In addition, note that the grain size and grain boundaries of the samples can also play a role in producing the abnormal change in resistivity. The reason why only sample x = 0.7 shows semiconductor-like transport behavior can also be addressed based on negative thermal expansion. The linear coefficient of NTE of sample x = 0.7 is smaller compared to other samples, as shown in Figure 5. Therefore, we believe that gradual change of lattice parameter as a function of temperature is a key ingredient for the occurrence of semiconductor-like feature. 

Another marked feature is the appearance of electrical resistivity minima below 50 K in the Mn_3_Zn_1−x_Co_x_N (x = 0.4, 0.7, 0.9) samples, which reflects the involvement of additional scattering factors. Generally, besides the well-known Kondo mechanism [30,31], there are other possible models that could account for the ρ minima, such as e-e interaction and electron-phonon interactions. In a strong electron-correlated system, the e-e interaction should play an important role in the electronic transport. To make a quantitative analysis, taking into account these mechanisms for minimum resistivity, the following equation was therefore taken to fit the low temperature resistivity data [30,31,32,33]

(1) ρ = A + BT12 − ClnT + DT5

where the coefficients A, B, C, and D represent the contributions from the residual resistivity, electron-electron (e-e) interactions, Kondo-like spin-dependent scattering, and electron-phonon (e-p) interactions, respectively. The curves are fitted well with Equation (1), as shown in the inset of Figure 4. The fitting parameters are listed in Table 2. The coefficient D related to e-p interactions is much smaller than B and C and can be neglected, suggesting that the behavior of electrical resistivity minima is mostly determined by the Kondo-like scattering and the e-e interactions. The coefficient B decreases as the Co concentration increases, indicating the suppression of the e*-*e scattering. This suggests that the appearance of the FM state may constrain the local spin directions and suppress the e-e scattering [33]. Because the Kondo-like scattering plays only a minor role, the good agreement between the experimental and fitted result suggests that the e-e interactions should be mainly responsible for the electrical resistivity minima. Even though the phenomenological fitting provides a reasonable explanation for the occurrence of resistivity minimum, other possibilities of generating such phenomenon such as chemical disorder or defects cannot be ruled out.

## 4. Conclusions

In summary, Mn_3_Zn_1−x_Co_x_N (x *=* 0.2, 0.4, 0.5, 0.7, 0.9) were synthesized by solid-state reaction in vacuum. The effect of Co doping on the magnetic, thermal expansion and resistivity properties of antiperovskite Mn_3_Zn_1−x_Co_x_N compounds was investigated. As a consequence of the doping of Co at the Zn site, all samples show competition between AFM and FM interactions, which are associated with the observed unusual electrical transport and negative thermal expansion behavior. The Mn_3_Zn_1−x_Co_x_N (x *=* 0.2, 0.4, 0.7, 0.9) compounds exhibit an abrupt resistivity jump phenomenon near the magnetic phase transition, which originates from the shift of the Fermi surface triggered by negative thermal expansion. Resistivity minima at low temperatures were observed in Mn_3_Zn_1−x_Co_x_N (x *=* 0.4, 0.7, 0.9) and the e-e interaction is likely responsible. 

## Figures and Tables

**Table 1:** Parameters obtained by the fitting of Curie-Weiss of Mn_3_Zn_1−x_Cu_x_N compounds.

Co (x)	Weiss Temperature (K)	Effective Moment μ_eff_ (μ_B_)
0.2	200	2.00
0.4	115	2.14
0.5	−22	2.76
0.7	−220	3.41
0.9	−380	3.68

**Table 2:** **Table 2** Parameters used to fit the resistivity data (H = 0 kOe) of the Mn_3_Zn_1-x_Co_x_N (x = 0.4, 0.7 and 0.9) compound.

x	A	B	C	D
0.2	-	-	-	-
0.4	1.69 × 10^−4^	6.69 × 10^−8^	9.06 × 10^−7^	9.95 × 10^−16^
0.7	1.02 × 10^−4^	5.41 × 10^−8^	1.96 × 10^−7^	1.49 × 10^−15^
0.9	7.14 × 10^−5^	2.31 × 10^−8^	2.43 × 10^−7^	1.14 × 10^−15^

## Supplementary Materials

The following are available online at http://www.mdpi.com/1996-1944/11/2/286/s1, Figure S1: Magnetoresistance curves measured at 5 K for samples with x = 0.4 and 0.7. No magnetoresistance phenomenon was observed.

## References

[1] Wang C., Chu L.H., Yao Q.R., Sun Y., Wu M.M., Ding L., Yan J., Na Y.Y., Tang W.H., Li G. (2012). Tuning the range, magnitude, and sign of the thermal expansion in intermetallic Mn_3_(Zn, *M*)_x_N(*M*= Ag, Ge). Phys. Rev. B.

[2] Takenaka K., Takagi H. (2005). Giant negative thermal expansion in Ge-doped anti-perovskite manganese nitrides. Appl. Phys. Lett..

[3] Song X.Y., Sun Z.H., Huang Q.Z., Rettenmayr M., Liu X., Seyring M., Li G., Rao G., Yin F. (2011). Adjustable zero thermal expansion in antiperovskite manganese nitride. Adv. Mater..

[4] Chu L.H., Wang C., Yan J., Na Y.Y., Ding L., Sun Y., Wen Y. (2012). Magnetic transition, lattice variation and electronic transport properties of Ag-doped Mn_3_Ni_1−x_Ag_x_N antiperovskite compounds. Scr. Mater..

[5] Lin S., Wang B.S., Lin J.C., Huang Y.N., Lu W.J., Zhao B.C., Tong P., Song W.H., Sun Y.P. (2012). Tunable room-temperature zero temperature coefficient of resistivity in antiperovskite compounds Ga_1−x_CFe_3_ and Ga_1−y_Al_y_CFe_3_. Appl. Phys. Lett..

[6] Ding L., Wang C., Chu L.H., Yan J., Na Y.Y., Huang Q.Z., Chen X. (2011). Near zero temperature coefficient of resistivity in antiperovskite Mn_3_Ni_1‒x_Cu_x_N. Appl. Phys. Lett..

[7] Asano K., Koyama K., Takenaka K. (2008). Magnetostriction in Mn_3_CuN. Appl. Phys. Lett..

[8] Huang R.J., Li L.F., Wu Z., Chu X., Xu X., Qian L. (2010). Spin-glass behavior in the antiperovskite manganese nitride Mn_3_CuN codoped with Ge and Si. Solid State Commun..

[9] Lin S., Shao D.F., Lin J.C., Zu L., Kan X.C., Wang B.S., Huang Y.N., Song W.H., Lu W.J., Tong P. (2015). Spin-glass behavior and zero-field-cooled exchange bias in a Cr-based antiperovskite compound PdNCr_3_. J. Mater. Chem. C.

[10] Ding L., Wang C., Sun Y., Colin C.V., Chu L.H. (2015). Spin-glass-like behavior and negative thermal expansion in antiperovskite Mn_3_Ni_1‒x_Cu_x_N compounds. J. Appl. Phys..

[11] Yan J., Sun Y., Wu H., Huang Q.Z., Wang C., Shi Z., Deng S.H., Shi K.W., Lu H., Chu L.H. (2014). Phase transitions and magnetocaloric effect in Mn_3_Cu_0.89_N_0.96_. Acta Mater..

[12] Lin S., Wang B.S., Lin J.C., Zhang L., Hu X.B., Huang Y.N., Lu W.J., Zhao B.C., Tong P., Song W.H. (2011). Composition dependent-magnetocaloric effect and low roomtemperature coefficient of resistivity study of iron-based antiperovskite compounds Sn_1‒x_Ga_x_CFe_3_ (0 ≤ x ≤ 1.0). Appl. Phys. Lett..

[13] Jardin J.P., Labbe J. (1983). Phase Transitions and Band Structure in Metallic Perovskites (Carbides and Nitrides). J. Solid State Chem..

[14] Garcia A.B.J., Marcelli A., Davoli I., Bartolome J. (1986). Local Electronic Structures at Selected Sites of Intermetallic perovskites Mn_3_MeX (Me=divalent metal, X = N, C). Il Nuovo Cim. D.

[15] Fruchart D., Bertaut E.F., Madar R., Fruchart R. (1971). Diffraction neutronique de Mn_3_ZnN. J. Phys. Coll..

[16] Deng S.H., Sun Y., Wang L., Shi Z., Wu H., Huang Q.Z., Yan J., Shi K.W., Hu P., Zaoui A. (2015). Frustrated Triangular Magnetic Structures of Mn_3_ZnN: Applications in Thermal Expansion. J. Phys. Chem. C.

[17] Sun Y.S., Guo Y.F., Wang X.X., Tsujimoto Y., Matsushita Y., Shi Y.G., Wang C., Belik A.A., Yamaura K. (2012). Resistive switching phenomenon driven by antiferromagnetic phase separation in an antiperovskite nitride Mn_3_ZnN. Appl. Phys. Lett..

[18] Sun Y., Wang C., Huang Q., Guo Y., Chu L.H., Arai M., Yamaura K. (2012). Neutron Diffraction Study of Unusual Phase Separation in the Antiperovskite Nitride Mn_3_ZnN. Inorg. Chem..

[19] Chu L.H., Wang C., Bordet P., Colin C.V., Pairis S., Na Y., Yan J., Huang Q. (2013). The effect of Zn vacancies on the physical properties of antiperovskite compounds Mn_3_Zn_x_N. Scr. Mater..

[20] Sun Y., Wang C., Wen Y., Zhu K., Zhao J. (2007). Lattice contraction and magnetic and electronic transport properties of Mn_3_Zn_1−x_Ge_x_N. Appl. Phys. Lett..

[21] Qu B.Y., Pan B.C. (2010). Nature of the negative thermal expansion in antiperovskite compound Mn_3_ZnN. J. Appl. Phys..

[22] Sun Y., Wang C., Wen Y., Chu L.H., Pan H., Nie M., Tang M. (2010). Negative Thermal Expansion and Magnetic Transition in Anti-Perovskite Structured Mn_3_Zn_1‒x_Sn_x_N Compounds. J. Am. Ceram. Soc..

[23] Tan J., Huang R., Li W., Han Y., Li L. (2014). Broadened negative thermal expansion operation-temperature window in antiperovskite Mn_3_Zn_0.6_Ge_0.4_N prepared by spark plasma sintering. J. Alloys Compd..

[24] Zhang X.H., Yin Y., Yuan Q., Han J.C., Zhang Z.H., Jian J.K., Zhao J.G., Song B. (2014). Magnetoresistance reversal in antiperovskite compound Mn_3_Cu_0.5_Zn_0.5_N. J. Appl. Phys..

[25] Lin J.C., Wang B.S., Tong P., Lin S., Lu W.J., Zhu X.B., Yang Z.R., Song W.H., Dai J.M., Sun Y.P. (2011). Tunable temperature coefficient of resistivity in C- and Co-doped CuNMn_3_. Scr. Mater..

[26] Chu L.H., Wang C., Sun Y., Li M.C., Wan Z.P., Wang Y., Dou S.Y., Chu Y. (2015). Doping Effect of Co at Ag Sites in Antiperovskite Mn_3_AgN Compounds. Chin. Phys. Lett..

[27] Roisnel T., Rodríguez-Carvajal J. (2001). WinPLOTR: A Windows Tool for Powder Diffraction Pattern Analysis. Mater Sci. Forum.

[28] Motizuki K., Nagai H. (1988). Electronic band structures and magnetism of the cubic perovskite-type manganese compounds Mn_3_MC (M = Zn, Ga, In, Sn). J. Phys. C Solid State Phys..

[29] Li B.Y., Li W., Feng W., Zhang Y., Zhang Z. (2005). Magnetic, transport and magnetotransport properties of Mn_3+x_Sn_1‒x_C and Mn_3_Zn_y_Sn_1‒y_C compounds. Phys. Rev. B.

[30] Abhay N.P., Radoslaw C.B., Jan M., Jacob E.G., Luke A.K.D., Paul L.M., Daniel C.R. (2004). The Kondo Effect in the Presence of Ferromagnetism. Science.

[31] Rana D.S., Markna J.H., Parmar R.N., Kuberkar D.G., Raychaudhuri P., John J., Malik S.K. (2005). Low-temperature transport anomaly in the magnetoresistive compound (La_0.5_Pr_0.2_)Ba_0.3_MnO_3_. Phys. Rev. B.

[32] Sun Y., Guo Y., Tsujimoto Y., Yang J., Shen B., Yi W., Matsushita Y., Wang C., Wang X., Li J. (2013). Carbon-Induced Ferromagnetism in the Antiferromagnetic Metallic Host Material Mn_3_ZnN. Inorg. Chem..

[33] Zhang J., Xu Y., Cao S., Cao G., Zhang Y., Jing C. (2005). Kondo-like transport and its correlation with the spin-glass phase in perovskite manganites. Phys. Rev. B.

