# Supplementary material for: Unusual Electrical Transport Driven by the Competition between Antiferromagnetism and Ferromagnetism in Antiperovskite Mn3Zn1−xCoxN
Source: Materials (Basel). 2018 Feb 12;11(2):286. doi: 10.3390/ma11020286 (PMC5848983; doi:10.3390/ma11020286)
Supplement: Supplementary file 1 [file materials-11-00286-s001.docx]

Supplementary

Unusual Electrical Transport Driven by the Competition between Antiferromagnetism and Ferromagnetism in Antiperovskite Mn_3_Zn_1−x_Co_x_N

Lihua Chu ^1,^*, Lei Ding ^2,^*, Cong Wang ^3^, Meicheng Li ^1^, Yanjiao Guo ^1^ and Zhuohai Liu ^1^

^1^ State Key Laboratory of Alternate Electrical Power System with Renewable Energy Sources, School of Renewable Energy, North China Electric Power University, Beijing 102206,
China; mcli@ncepu.edu.cn (M.L.); guoxiaojiaooo@163.com (Y.G.); zhuohaiLiu666@163.com (Z.L.)

^2^ ISIS Facility, Rutherford Appleton Laboratory, Harwell Oxford, Didcot OX11 0QX, UK

^3^ Center for Condensed Matter and Materials, Department of Physics, Beihang University, Beijing 100191, China; congwang@ncepu.edu.cn

***** Correspondence: lhchu@ncepu.edu.cn (L.C.); lei.ding@stfc.ac.uk (L.D.); Tel.: +86-10-61772332 (L.C.);
+44-(0)1235-445093 (L.D.)

Received: 5 January 2018; Accepted: 9 February 2018; Published: date

**Supplementary Materials**

**Figure S1** Magnetoresistance curves measured at 5K for samples with x=0.4 and 0.7. No magnetoresistance phenomenon was observed.

© 2018 by the authors. Submitted for possible open access publication under the
terms and conditions of the Creative Commons Attribution (CC BY) license (http://creativecommons.org/licenses/by/4.0/).
